# Cocaine‐related vasculitis

**DOI:** 10.1002/ccr3.3450

**Published:** 2020-10-26

**Authors:** Hassan Liaqat, Narine Shirvanian, Mohammad Ammad Ud Din, Ali Amin

**Affiliations:** ^1^ Department of Internal Medicine Henry Ford Allegiance Health Jackson MI USA; ^2^ Department of Internal Medicine Rochester General Hospital Rochester NY USA; ^3^ Department of Allergy, Immunology and Rheumatology Henry Ford Allegiance Jackson MI USA

**Keywords:** cocaine use, emergency medicine, substance abuse, toxicology, vasculitis

## Abstract

Patients presenting with pancytopenia and a painful purpuric rash should be evaluated for levamisole‐induced vasculitis and counseled about cocaine cessation as continued exposure can lead to permanent deformity of the involved areas.

## Graphical abstract

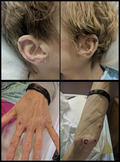

A 55‐year‐old woman with a history of illicit substance use presented with joint pains and ulceration of the skin on the earlobes and elbows. Laboratory evaluation revealed pancytopenia and positive c and p‐ANCA. She was diagnosed with levamisole‐induced vasculitis. Her condition improved with steroids and cessation of cocaine use.

A 55‐year‐old woman with a history of cocaine use presented with arthralgias and painful nonspecific skin ulcers. The physical examination was notable for a painful purpuric rash with central necrosis on the ear lobes, bridge of the nose, and bilateral distal upper extremities (Figure 1). Laboratory analysis revealed pancytopenia; white blood cell count 2900/uL, hematocrit 29%, and platelets 94 000/uL as well as elevated titers of cytoplasmic antineutrophil cytoplasmic antibodies (c‐ANCA), perinuclear antineutrophil cytoplasmic antibodies (p‐ANCA) with positive proteinase‐3. Given the characteristic distribution of the rash along with the positive ANCA antibodies, the diagnosis of levamisole‐induced vasculitis was established.

**FIGURE 1:**
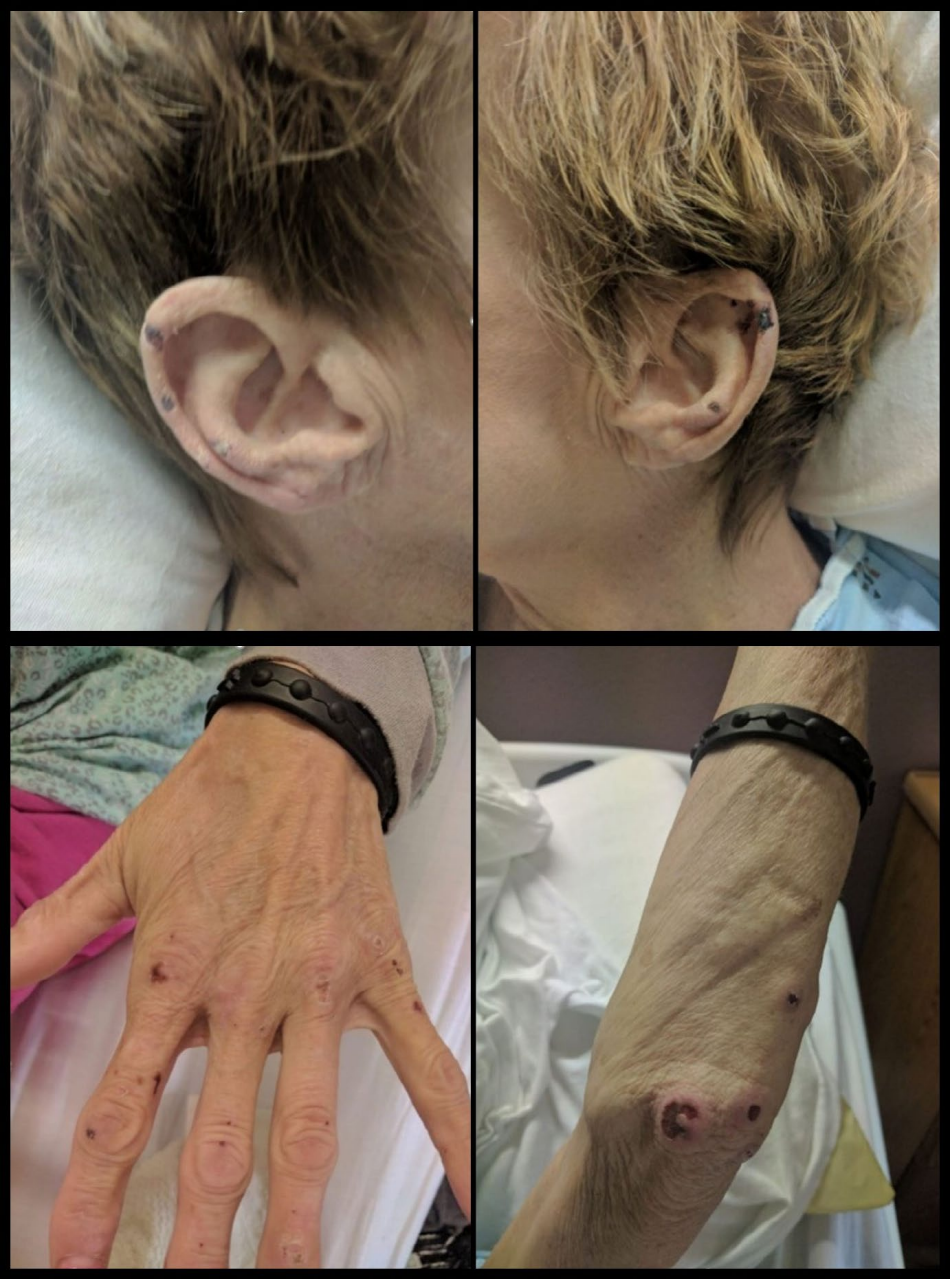
Purpuric lesions with central necrosis seen on both earlobes (top panels), digits of the left upper extremity (bottom left panel), and the left elbow (bottom right panel) consistent with levamisole‐induced vasculitis

A large proportion of cocaine sold is adulterated with levamisole as it inhibits catechol O‐methyltransferase and monoamine oxidase activity, and thus, potentiates cocaine's reuptake inhibition.^1^ The adverse effects of adulterated cocaine primarily involve agranulocytosis and vasculitis and are seen both with smoking and snorting cocaine.^2^ Lesions involve the ears, nose, cheeks, and extremities. They start as tender purpura, progressing to bullae with eventual necrosis and eschar formation.^2^ Most cases resolve spontaneously over months after cocaine cessation as the acute inflammation resolves.^2^ The patient was treated with steroids was referred to an outpatient chemical dependency program.

## CONFLICT OF INTEREST

The authors declared no potential conflicts of interest with respect to the research, authorship, and/or publication of this manuscript.

## AUTHOR CONTRIBUTIONS

HL and MAUD: Authors were involved in writing of the entire manuscript. NS: Conceptualized the manuscript and provided the images for the manuscript. AA: Primary rheumatologist on the case and critically reviewed the manuscript and made final edits prior to the submission.

## ETHICAL APPROVAL

This case report was conducted in accordance with the Declaration of Helsinki. The collection and evaluation of all protected patient health information were performed in a Health Insurance Portability and Accountability (HIPAA) complaint manner.
